# Presurgical management of ulnar nerve entrapment in patients with and without diabetes mellitus

**DOI:** 10.1038/s41598-024-66592-y

**Published:** 2024-07-06

**Authors:** Stina Andersson, Malin Zimmerman, Raquel Perez, Mattias Rydberg, Lars B. Dahlin

**Affiliations:** 1 Department of Translational Medicine – Hand Surgery, Lund University, Malmö, Sweden; 2 Department of Hand Surgery, Skåne University Hospital, Malmö, Sweden; 3 Department of Orthopedics, Helsingborg Hospital, Helsingborg, Sweden; 4 Unit for Social Epidemiology, Department of Clinical Sciences (Malmö), Faculty of Medicine, Lund University, 20502 Malmö, Sweden; 5 Department of Biomedical and Clinical Sciences, Linköping University, Linköping, Sweden

**Keywords:** Peripheral neuropathies, Diabetes complications

## Abstract

Diabetes mellitus (DM) is a risk factor for the development of ulnar nerve entrapment (UNE). Differences in surgical outcomes for UNE between patients with and without DM have been reported, but studies on presurgical management are scarce. This study aimed to evaluate the presurgical management of UNE in patients with (DM diagnosis < 2 years of UNE diagnosis) and without DM regarding the level of care for diagnosis and the proportion that proceeds to surgery. Data from 6600 patients with UNE between 2004 and 2019 were included from the Skåne Health Care Register (SHR) and cross-linked with data from the Swedish National Diabetes Register (NDR). The group with UNE and DM was compared to the group with UNE without DM and prevalence ratios were calculated using Cox regression analysis. Patients with DM more often got their first UNE diagnosis in secondary care than in primary care (643/667, 96% vs. 5361/5786; 93%). Patients with and without DM, regardless of the type of DM, presence of retinopathy, or HbA1c levels, were surgically treated for UNE to the same extent (258/667, 39% of patients with DM vs. 2131/5786, 37% of patients without DM). The proportion of surgically treated was significantly lower among patients diagnosed with UNE in primary care compared to secondary care (59/449, 13% vs. 2330/5786, 38%). Men were more often surgically treated than women (1228/3191, 38% vs.1161/3262 36%). Patients with UNE and DM are surgically treated to the same extent as patients with UNE but without DM and are more likely to be diagnosed in specialized care.

## Introduction

Ulnar nerve entrapment (UNE) at the elbow is the second most common nerve compression in the upper extremity after carpal tunnel syndrome (CTS)^1^, with an incidence of around 20–30 cases per 100,000 person-years^2,3^. Risk factors for UNE include older age, male sex, smoking, manual work, and diabetes mellitus (DM)^4–8^. The mechanism(s) that cause this increased risk of UNE in DM is not yet completely understood; however, hyperglycemia negatively affects the peripheral nerve in several ways.

Age, male sex, and diabetic retinopathy are risk factors for developing peripheral nerve entrapment disorders in the hand/upper limb^9^. A recently published study on nerve entrapment disorders in patients with DM showed that both CTS and UNE were more common in patients with diabetic retinopathy than without, and higher HbA1c levels, longer diabetes duration, and higher BMI were also significant risk factors for the development of CTS/UNE^10^.

For patients with DM, UNE may be present without clinical symptoms and just be observed as subclinical electrophysiological alterations^11,12^. A proposed theory is that the threshold of the sensory nerves is altered in diabetes, which lowers the propensity for these patients to develop a clinical symptomatology^13^.

There is no consensus in the literature on indications for surgery or which patients benefit from which treatment strategy. Therefore, the treatment choice for UNE is mainly based on the surgeon’s or the healthcare center’s preferences and experiences^14–16^. Higher revision rates for patients with DM following surgery for UNE have been presented^17^. However, evaluations of patient-reported outcomes for surgically treated patients with UNE have not shown any general differences for patients with and without DM^18–20^.

The clinical management of different patient groups has lately received attention for other hand surgical conditions and affects health care quality and equality^21,22^. A delay in diagnosis and treatment of UNE may decrease the likelihood of full recovery due to the risk of remaining injuries to the nerve trunk, particularly the nerve fibers, i.e., both the axons and the Schwann cells, even after the surgery^23,24^. Since UNE in patients with DM seems to have a different pathophysiology, with an already damaged and more vulnerable peripheral nerve, compared to patients without DM, the clinical management and surgical approaches between patients with and without DM may differ.

This study aimed to investigate the presurgical clinical management of patients with UNE, including the level of care for diagnosis, the proportion of surgically treated patients, and whether this course differs between patients with and without DM.

## Methods

### Study design

The study is a retrospective observational study based on data from two Swedish registers, the Skåne Health Care Register (SHR) and the Swedish National Diabetes Register (NDR), between 2004 and 2019. SHR includes data on patients with a UNE diagnosis (according to ICD-10 SE codes) in the health care Region of Skåne in Southern Sweden with additional information as age, sex, anatomic level of the nerve entrapment (based on ICD-10 SE codes), date of diagnosis, level of care for the diagnosis and eventual surgeries performed. The register has been previously validated by several studies^25,26^.

NDR is a Swedish national quality register started in 1996 for patients with diabetes, including a variety of variables such as age, sex, the debut year of diabetes, type of diabetes, diabetes treatment, presence of retinopathy, and continuously measured parameters such as blood pressure, body mass index (BMI), glomerular filtration rate (GFR), creatinine, HbA1c, and blood lipids. Both primary care and specialized care report data to NDR, and the register covers around 85–90% of all patients with DM in Sweden^27–29^. Patients identified with a documented UNE in SHR, defined by the ICD-10 SE codes G562, G562C, and G562D, were included in the study, and data was cross-linked with NDR to determine diabetes status using the Swedish unique personal identification numbers. Patients < 18 years at the time of UNE diagnosis and those with DM other than type 1 or 2 were excluded from the study. Only diagnoses in somatic specialized care and primary care were included. All patients provided written informed consent before inclusion in NDR. Laboratory data was only available for the patients with diabetes since it is only registered in the NDR and not in the SHR.

### Study groups

The UNE patients were divided into two main groups, i.e., patients with DM and patients without. Patients with DM were defined as DM diagnosis before or within two years of the time of UNE diagnosis. Patients with an onset of DM more than 2 years after the time for the first UNE diagnosis were designated into the group without DM. If data on the debut date of a patient’s DM was missing, the date for the first registered visit in the NDR was used. HbA1c was analyzed and divided into tertiles according to the patient’s mean value of all registered HbA1c levels during the study period. The HbA1c tertiles were defined as optimal control (HbA1c ≤ 51.1 mmol/mol), acceptable control (HbA1c 51.2–63.7 mmol/mol), and poor glycemic control (> 63.7 mmol/mol) in line with the American Diabetes Association guidelines for glycemic targets^30^.

### Level of care

The level of care was defined as the healthcare level where a patient had their first documented UNE diagnosis: primary care, secondary specialized outpatient care, and psychiatric care. Only one patient was diagnosed in psychiatric care, and this case was excluded from the statistical analysis.

### Surgical treatments

Surgical treatments for UNE in the SHR included simple decompressions (ICD-10 ACC53) and transposition surgeries (ICD-10 ACC43). The variable ‘surgically treated’ was defined as a dichotomous nominal variable. If yes, the patient was surgically treated at least once for UNE at any level (i.e., any of the included ICD-10 diagnostic codes) with any of the two surgical procedures.

### Statistics

Descriptive statistics are presented as mean ± standard deviation (SD) continuous variables. Nominal variables are presented as numbers and percent, n (%).

Since the prevalence of surgical treatment was relatively high, the association between diabetes and surgical treatment was presented as prevalence ratios (PRs) rather than odds ratios. For this purpose, we applied Cox regression with constant time at risk^31^. We performed two regression models. The unadjusted model included only diabetes status, and the adjusted model added demographic and patient characteristic variables (i.e., sex, age, and level of care).

Then, we developed two regression models only in patients with diabetes. The unadjusted model included only type of diabetes and the adjusted model add demographic, patient characteristic and clinical measurement (i.e., sex, age, level of care, retinophaty, duration of diabetes, body mass index, glycemic control, creatinine and cholesterol levels).

Stata v14.1 (StataCorp, College Station, TX) was used to conduct the analyses.

### Ethics

The Swedish Ethical Review Authority (Etikprövningsmyndigheten) approved this study (Dnr: 2019-02042; 2021-02029). The study was conducted in accordance with the ethical considerations stated in the Declaration of Helsinki.

## Results

### Characteristics

**Figure 1:**
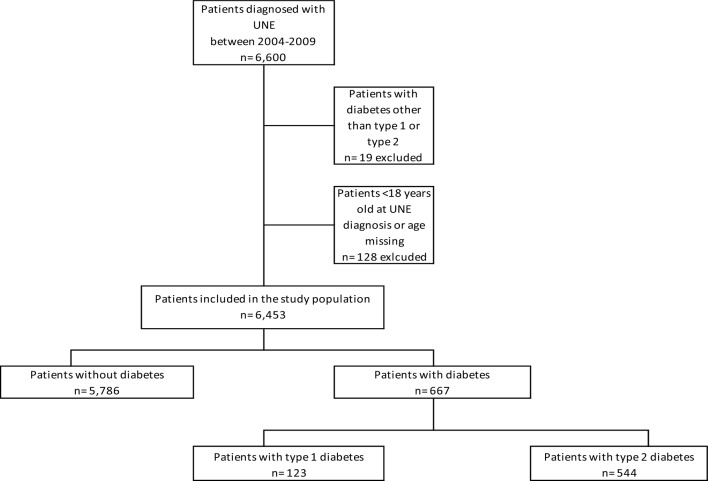
Flowchart of the included study population with ulnar nerve entrapment (UNE), with and without diabetes, from Skåne Health Care Register (SHR) and the Swedish National Diabetes Register (NDR) between 2004 and 2019. *UNE* ulnar nerve entrapment at any level. Excluded patients include patients < 18 years at UNE diagnosis, patients with diabetes other than type 1 or type 2, and one patient diagnosed with diabetes in psychiatric care. The type of diabetes was missing in 10 patients. These were included in analyses with the total diabetes population, but not in sub-analyses when type of diabetes was used.

Data from 6600 patients with UNE were reported in SHR from 2004 to 2019. We excluded 128 patients due to age < 18 years by the time of the first UNE diagnosis and 19 patients with diabetes other than type 1 or 2 (Fig. 1). Among the included 6,453 patients in the study population, the mean age at UNE diagnosis was 51 ± 15 years, and 3131/6600 (50%) were men. During the study period, 7817 individual diagnoses of UNE were reported, i.e., some patients had more than one documented diagnosis. UNE at the elbow level (ICD-10 G562C) was most common (3872/7817, 50%), followed by undefined lesion of the ulnar nerve (ICD-10 G562; 2837/7817, 36%) and UNE at the wrist (ICD-10 G562D; 1108/7817, 14%). In the study population, 667/6453 (10%) patients were also registered in the Swedish National Diabetes Register (NDR) and had a diabetes diagnosis. Of these, 608 patients had established diabetes before their first UNE diagnosis or within two years of the time of UNE diagnosis and were defined as the diabetes group (Fig. 1).

### Characteristics of the group with diabetes

The patients in the group with DM were older and more often men than those in the group without DM (Table 1). In the group with DM, 123/6453 (18%) had type 1 diabetes, and 544/6453 (9%) patients had type 2 diabetes (Fig. 1, Table 1). Data on DM type was missing in ten cases. Diabetic retinopathy was present at baseline in 297/667 (47%) of patients.

**Table 1:** Descriptive characteristics of patients with ulnar nerve entrapment (UNE) at any level and registered in Skåne Health Care Register (SHR), combined with data on diabetes from the Swedish National Diabetes Register (NDR) during 2004–2019.

	No diabetes	Diabetes		
		Type I	Type II	Total
	5786 (89.66)	123 (1.91)	544 (8.43)	667 (10.34)
Age (years)				
18–44	2216 (38.3)	43 (35.0)	42 (7.7)	85 (12.7)
45–64	2583 (44.6)	63 (51.2)	269 (49.5)	332 (49.8)
> = 65	987 (17.1)	17 (17.1)	233 (42.8)	250 (37.5)
Sex				
Men	2797 (48.3)	73 (55.73)	497 (60.61)	394 (59.1)
Women	2989 (51.7)	58 (44.27)	323 (39.39)	273 (40.9)
Surgically treated				
No	3655 (63.2)	80 (65.0)	329 (60.5)	409 (61.3)
Yes	2131 (36.8)	43 (35.0)	215 (39.2)	258 (38.7)
Level of care				
Primary care	425 (7.4)	2 (1.6)	22 (4.0)	24 (3.6)
Specialist care	5361 (92.7)	121 (98.4)	522 (96.0)	643 (96.4)
Diabetic retinopathy				
No		27 (22.1)	309 (60.5)	336 (53.1)
Yes		95 (77.9)	202 (39.5)	297 (46.9)
BMI (kg/m^2^)		26.8 ± 4.3	31.5 ± 5.2	30.6 ± 5.4
Glycemic control				
Optimal		12 (9.8)	217 (38.9)	229 (34.3)
Acceptable		46 (37.4)	178 (32.7)	224 (33.6)
Poor		65 (52.9)	149 (27.4)	214 (32.1)
Creatinine (µmol/l)		88.7 ± 64.0	85.0 ± 35.6	85.7 ± 42.3
Diabetes duration (years)		35.0 ± 14.3	14.7 ± 8.7	18.5 ± 12.7
GFR		86.8 ± 25.1	80.3 ± 23.3	81.5 ± 23.8

Values are presented as n (%) and mean ± standard deviation, SD. Patients with diabetes include patients who had their diabetes diagnosis before their UNE diagnosis. Patients with a diabetes debut after a documented UNE were categorized as “without diabetes”.

### Level of care for diagnosis

The majority of the study population, 6004/6453 (93%), had a first-time diagnosis in secondary care, and 449/6543 (7%) were diagnosed in primary care (Table 2). Patients without DM were more often diagnosed in primary care compared to patients with DM (Table 1). The level of care for diagnosis did not differ among the group with DM depending on the type of DM or the presence of retinopathy (Table 2). The group with optimal HbA1c control was to a greater extent diagnosed in specialized care than the group with poor HbA1c control (Table 2).

**Table 2:** Proportion diagnosed in primary care and proportion of surgically treated patients with ulnar nerve entrapment (UNE) at any level and registered in the Skåne Health Care Register (SHR) and the Swedish National Diabetes Register (NDR) during 2004–2019.

	Level of care (primary care)	Surgically treated
All patients	449 (7.0)	2389 (37.0)
Men	229 (7.2)	1228 (38.5)
Women	220 (6.7)	1161 (35.6)
No diabetes	425 (7.4)	2131 (36.8)
Diabetes	24 (3.6)	258 (38.7)
Type 1	2 (1.6)	43 (35.0)
Type 2	22 (4.0)	215 (39.5)
Glycemic control		
Optimal	13 (5.6)	88 (38.4)
Acceptable	8 (3.6)	82 (36.6)
Poor	3 (1.4)	88 (41.1)
Retinopathy	8 (2.7)	131 (39.0)
No retinopathy	15 (4.5)	118 (39.7)
Level of care		
Primary care		59 (13.1)
Specialist care		2330 (38.8)

Values presented as n (%). Level of care refers to where the patients had their first UNE diagnosis. Surgically treated patients include patients with at least one registered surgical procedure for UNE at any level during the study period. Glycemic control is defined as optimal (HbA1c ≤ 51.1 mmol/mol), acceptable (HbA1c 51.2–63.7 mmol/mol), and poor (HbA1c > 63.7 mmol/mol).

### Surgical treatment

In total, 2389 surgical procedures for UNE were performed and documented in the SHR during the study period. Of these, 290/2389 (12%) were transposition surgeries (ICD-10 ACC43), and 2099/2389 (93%) were nerve decompressions (ICD-10 ACC53). In the study population, 2389/6600 (37%) patients had at least one documented surgery for UNE at any anatomical level. The distribution of performed surgical treatment, i.e., nerve decompression or transposition surgery, was the same in patients with DM and without DM. Men were more often surgically treated than women: 1228/2389 (38%) men compared to 1161/2389 (36%) women. The proportion of surgically treated patients did not differ between patients with and without DM (Table 2). Among the group with DM, the proportion of surgically treated patients did not significantly differ between patients with type 1 and type 2 DM or between patients with diabetic retinopathy and without retinopathy (Table 2). Patients diagnosed with UNE in the secondary care were more often surgically treated than patients initially diagnosed in the primary care (Table 2). The proportion of surgically treated patients did not differ between groups according to glycemic control, i.e., HbA1c levels (Table 2). In the Cox regression analysis, neither type 1 nor type 2 DM increased the likelihood of surgical treatment (adjusted model prevalence ratio PR 0.90 (95% CI 0.66–1.22) for type 1 DM and 1.04 (0.90–1.20) for type 2 DM; Table 3). Men had a higher but not statistically significant risk of surgical treatment than women (PR 1.08 (95% CI 1.00–1.17); Table 3). The risk of surgical treatment was higher in individuals aged 45–64 years compared to individuals aged 18–44 years (PR 1.15 (1.05–1.26); Table 3). Individuals diagnosed in specialist care were more likely to receive surgical treatment (PR 2.92 (95% CI 2.26–3.78); Table 3).

**Table 3:** Cox regression model on the risk of surgical treatment in patients with ulnar nerve entrapment.

	Unadjusted	Adjusted
Diabetes		
No	Reference	Reference
Type 1	0.95 (0.70–1.28)	0.90 (0.66–1.22)
Type 2	1.07 (0.93–1.23)	1.04 (0.90–1.20)
Sex		
Women		Reference
Men		1.08 (1.00–1.17)
Age		
18–44		Reference
45–64		1.15 (1.05–1.26)
> = 65		0.96 (0.85–1.09)
Level of care		
Primary		Reference
Specialist		2.92 (2.26–3.78)

Numbers are prevalence ratios with 95% confidence interval.

When separately studying individuals with diabetes, the risk of surgical treatment was higher in individuals with type 2 DM compared to individuals with type 1 DM (PR 1.75 (1.07–2.87); Table 4). Longer duration of diabetes was marginally associated with a slightly elevated risk of surgical treatment (PR 1.01 (1.00–1.03); Table 4). Retinopathy, BMI, glycemic control, creatinine levels, and cholesterol levels did not affect the risk of surgical treatment in individuals with diabetes (Table 4).

**Table 4:** Cox regression analysis on the risk of surgical treatment of ulnar nerve entrapment in individuals with diabetes.

	Unadjusted	Adjusted
Diabetes		
Type 1	Reference	Reference
Type 2	1.13 (0.81–1.57)	1.75 (1.07–2.87)
Sex		
Women		Reference
Men		1.25 (0.94–1.65)
Age		
18–44		Reference
45–64		0.95 (0.61–1.47)
> = 65		0.73 (0.45–1.19)
Level of care		
Primary		Reference
Specialist		8.71 (1.22–62.25)
Retinopathy		
No		Reference
Yes		0.98 (0.73–1.32)
Duration of diabetes (years)		1.01 (1.00–1.03)
BMI (kg/m^2^)		0.99 (0.96–1.02)
Glycemic control		
Optimal		Reference
Acceptable		0.80 (0.58–1.12)
Poor		0.90 (0.63–1.27)
Cretatinine (µmol/l)		1.00 (1.00–1.00)
Cholesterol (mmol/L)		0.99 (0.86–1.14)

Numbers are prevalence ratios with 95% confidence interval. Glycemic control is defined as optimal (HbA1c ≤ 51.1 mmol/mol), acceptable (HbA1c 51.2–63.7 mmol/mol), and poor (HbA1c > 63.7 mmol/mol).

## Discussion

Diabetes is a known risk factor for a variety of peripheral neuropathies, including nerve entrapment disorders like UNE. In the present register-based retrospective study, patients with and without diabetes were surgically treated for UNE to the same extent. The proportion of surgically treated patients did not depend on HbA1c levels, type of DM, or the presence of retinopathy among the patients with DM. However, we did find that within the population with diabetes, individuals with type 2 DM were more likely to be surgically treated than individuals with type 1 DM. In a recent study, we found the opposite in carpal tunnel syndrome; individuals with type 1 DM were more likely to be surgically treated than individuals with type 2 DM^32^. These findings may reflect the pathoanatomical differences in CTS and UNE^33^, or perhaps a slightly different risk factor panorama in the different types of diabetes. We also hypothesized that a long duration of diabetes would be associated with a higher risk of surgical treatment due to the higher risk of neuropathy, but it was only borderline significant in the regression analysis. In previous studies, patients with DM have had equally good results of surgical procedures based on patient-reported outcomes compared to patients without DM, regardless of type of DM or the presence of retinopathy^18–20^. Based on these previous results, it can be assumed that patients with and without DM may benefit equally from surgical treatment. It is, therefore, reasonable that patients with and without DM should be surgically treated to the same extent, which is in line with the results of the present study. However, other aspects than patient-reported outcomes are also of interest and should be considered when evaluating surgical treatment. For example, higher revision rates have been presented for patients with DM following surgery for UNE^17^. Other possible predictors for surgery that may differ between patients with and without DM are preoperative electrophysical findings and symptom severity. More severe preoperative UNE has been associated with worse surgical outcomes^34,35^. When taking this into account, as well as the fact that patients with DM may more often have asymptomatic ulnar nerve entrapments, it would be valuable to include patients’ symptoms when analyzing the proportion of surgically treated patients.

Patients with UNE and a concomitant DM were less likely to be diagnosed in primary care, i.e., 4% were diagnosed in primary care compared to 7% among patients without diabetes. It can be assumed that due to diabetes, these patients already have established contact with specialized secondary care in some way and, therefore, have easier and faster access to secondary care regarding other health issues. UNE and its relation to DM may also be recognized more by physicians specializing in diabetes, making them more prone to diagnosing the condition. Of the patients diagnosed in primary care, only 12% were surgically treated, compared to 37% in the total study population. One explanation for this might be that individuals with a more severe UNE, possibly requiring surgery, are more often referred to specialist care, thus increasing the proportion of individuals undergoing surgery in specialist care. As mentioned, this study does not consider symptoms and UNE grading. It is possible that patients with UNE in primary care have milder symptoms and, therefore, do not receive or need surgical treatment.

An interesting finding, which was not a part of the original aim, was that men in this study were more often surgically treated for UNE than women, even though in the regression model, male sex did not reach statistical significance. Male sex is a risk factor for the development of UNE, but to our knowledge, no sex differences regarding surgical outcomes have been established. In a study evaluating patient-reported outcomes based on answers from questionnaires regarding physical function and symptoms (i.e., QuickDASH), women had more symptoms than men both pre-and postoperatively. However, the improvement in QuickDASH score after surgery was larger in women^18^. Based on that single study, one might hypothesize that women would benefit more from surgery and more often be surgically treated, which is not in line with the present study results. It is also possible that men present with more severe UNE^36^ or that surgeons are more likely to propose surgical treatment to male patients.

The fact that the study method is based on registries confers both strengths and limitations. One of the main strengths of this study is the large study population and the fact that the used registries include data from both primary and secondary care, as well as private and regional clinics, which makes the results applicable to the entire healthcare system. Many studies evaluating surgical outcomes only include patients who have already been surgically treated. With the present data, we could evaluate and focus on patients’ entire healthcare process from diagnosis to surgery. One limitation of this study is that the registries do not include factors such as symptoms and severity, electrophysiological examinations, and comorbidities, which would have contributed to additional important aspects in the analysis of all variables. There was also missing data on diabetic retinopathy status. Moreover, one of the used registries (SHR) is based on ICD-10 codes and hence has the limitation that the validity of the registry is dependent on how well and accurately the clinics apply the diagnostic coding.

## Conclusion

Patients with UNE and DM are surgically treated to the same extent as patients with UNE but without DM and are more likely to be diagnosed in specialized care.

## Data Availability

Data is not publicly available. Swedish law limits public access to the data used for this study. Researchers can request data after applying to the Swedish Ethical Review Authority.
